# The Role of Phyto-Melatonin and Related Metabolites in Response to Stress

**DOI:** 10.3390/molecules23081887

**Published:** 2018-07-28

**Authors:** Yang Yu, Yan Lv, Yana Shi, Tao Li, Yanchun Chen, Dake Zhao, Zhiwei Zhao

**Affiliations:** 1 State Key Laboratory for Conservation and Utilization of Bio-resources in Yunnan, Yunnan University, Kunming 650091, China; YuYang_YNU@163.com (Y.Y.); litao@ynu.edu.cn (T.L.); 2 School of Agriculture, Yunnan University, Kunming 650504, China; lvyanshj@163.com (Y.L.); lqhclucky@163.com (Y.C.); 3 Institute of Medicinal Plants, Yunnan Academy of Agricultural Sciences, Kunming 650205, China; bestshiyana@163.com; 4 Biocontrol Engineering Research Center of Plant Disease & Pest, Yunnan University, Kunming 650504, China; 5 Biocontrol Engineering Research Center of Crop Disease & Pest, Yunnan University, Kunming 650504, China

**Keywords:** melatonin, reactive oxygen species, biosynthesis, catabolism, stress resistance

## Abstract

Plant hormone candidate melatonin has been widely studied in plants under various stress conditions, such as heat, cold, salt, drought, heavy metal, and pathogen attack. Under stress, melatonin usually accumulates sharply by modulating its biosynthesis and metabolic pathways. Beginning from the precursor tryptophan, four consecutive enzymes mediate the biosynthesis of tryptamine or 5-hydroxytryptophan, serotonin, *N*-acetylserotonin or 5-methoxytryptamine, and melatonin. Then, the compound is catabolized into 2-hydroxymelatonin, cyclic-3-hydroxymelatonin, and *N*^1^-acetyl-*N*^2^-formyl-5-methoxyknuramine through 2-oxoglutarate-dependent dioxygenase catalysis or reaction with reactive oxygen species. As an ancient and powerful antioxidant, melatonin directly scavenges ROS induced by various stress conditions. Furthermore, it confreres stress tolerance by activating the plant’s antioxidant system, alleviating photosynthesis inhibition, modulating transcription factors that are involved with stress resisting, and chelating and promoting the transport of heavy metals. Melatonin is even proven to defense against pathogen attacks for the plant by activating other stress-relevant hormones, like salicylic acid, ethylene, and jasmonic acid. Intriguingly, other precursors and metabolite molecules involved with melatonin also can increase stress tolerance for plant except for unconfirmed 5-methoxytryptamine, cyclic-3-hydroxymelatonin, and *N*^1^-acetyl-*N*^2^-formyl-5-methoxyknuramine. Therefore, the precursors and metabolites locating at the whole biosynthesis and catabolism pathway of melatonin could contribute to plant stress resistance, thus providing a new perspective for promoting plant stress tolerance.

## 1. Introduction

Melatonin (*N*-acetyl-5-methoxytryptamine), which is widespread in almost all organisms, appears to be a multi-regulatory molecule with multiple functions in plant growth and development, such as seed germination, root development, fruit ripening, senescence, yield, circadian rhythm, and response to stress [1,2,3]. Under various abiotic and biotic stresses, such as heat, cold, salt, drought, heavy metal, and pathogen attack, melatonin usually could directly scavenge ROS generating from these stresses as a powerful antioxidant, thus promoting the stress resistance for plants [1,4,5,6,7]. Furthermore, melatonin could improve stress tolerance by activating the plant’s antioxidant system, alleviating photosynthesis inhibition, modulating transcription factors that are involved with stress resisting, chelating and promoting transport of heavy metals, or activating other stress-relevant hormones, like salicylic acid, ethylene, and jasmonic acid [8,9,10,11]. Therefore, phyto-melatonin (melatonin in plants) plays the key role in plant stress response.

It is well understood that the biosynthesis of phyto-melatonin begins with tryptophan and it involves several intermediates, including tryptamine, 5-hydroxytryptophan, serotonin, *N*-acetylserotonin, and 5-methoxytryptamine [12]. Thereafter, melatonin is converted into other metabolites, including 2-hydroxymelatonin, cyclic-3-hydroxymelatonin, or *N*^1^-acetyl-*N*^2^-formyl-5-methoxyknuramine (AFMK) [13,14]. Previous research showed that, besides melatonin, its precursors and metabolites also participated in the plant stress resistance (Table 1). Though these compounds that are involved in the synthesis and metabolism pathways of melatonin play a role in stress resistance [15,16], no review is available focusing on the complete biosynthesis and catabolism pathway of melatonin under various abiotic and biotic stresses. Herein, the role of phyto-melatonin and its precursors and metabolites in response to stress is reviewed, thus providing a new perspective for promoting plant stress resistance.

## 2. The Biosynthesis and Catabolism Pathway of Melatonin

**Figure 1:**
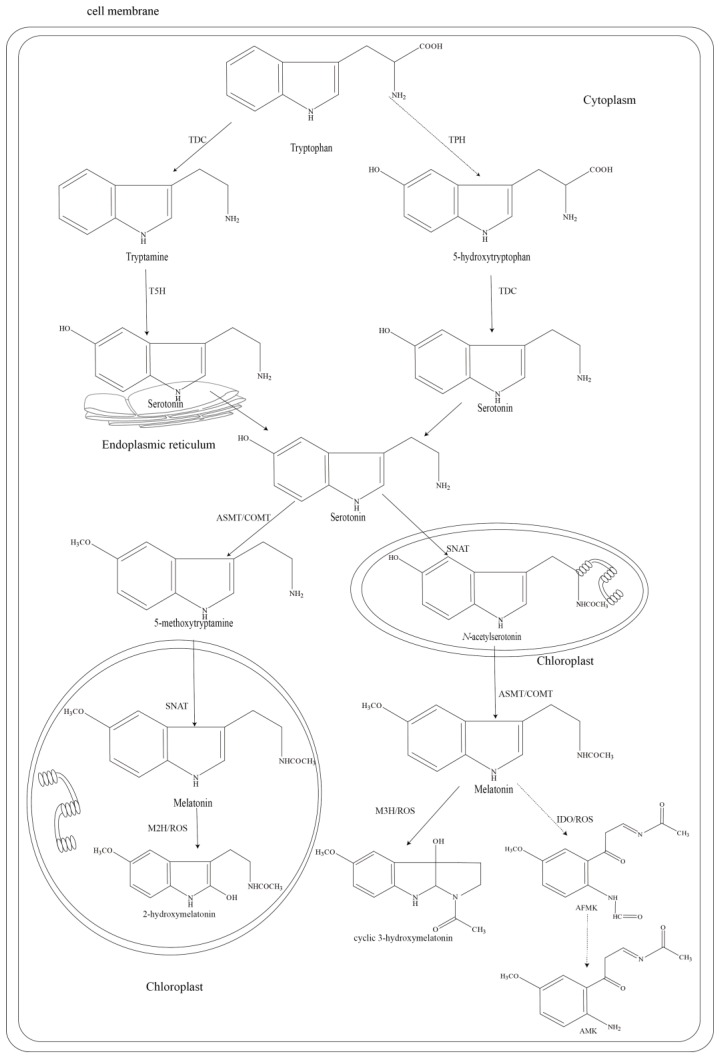
Melatonin biosynthesis and catabolism pathways in plants. Abbreviation: TDC, tryptophan decarboxylase; TPH, tryptophan hydroxylase; T5H, tryptamine 5-hydroxylase; SNAT, serotonin *N*-acetyltransferase; ASMT, *N*-acetylserotonin methyltransferase; COMT, caffeic acid *O*-methyltransferase; AFMK, *N*^1^-acetyl-*N*^2^-formyl-5-methoxyknuramine; AMK, *N*-acetyl-5-methoxyknuramine; M2H, melatonin 2-hydroxylase; M3H, melatonin 3-hydroxylase; IDO, indoleamine 2,3-dioxygenase; 2-ODD, 2-oxoglutarate-dependent dioxygenase; ROS, reactive oxygen species. Dotted arrows represent the hypothetical steps.

The biosynthetic pathway of phyto-melatonin has been elucidated recently (Figure 1). The molecule is produced via four consecutive enzymatic steps with tryptophan as the initial substrate and at least six enzymes are involved in melatonin synthesis, including TPH, TDC, T5H, SNAT, ASMT, and COMT [65]. Excluding TPH, genes encoding other five enzymes have been cloned [12,66,67,68,69]. The first enzymic step is the decarboxylation of tryptophan catalyzed by TDC to produce tryptamine in the cytoplasm, followed by the enzymatic hydroxylation by T5H to generate serotonin in the endoplasmic reticulum [70]. For serotonin synthesis, alternatively, tryptophan may first be hydroxylated by one TPH (un-identified) in the cytoplasm in order to generate 5-hydroxytryptophan, followed by decarboxylated with TDC to produce serotonin in the cytoplasm [71]. Afterwards, both SNAT and ASMT, through acetylation and methylation, respectively, convert the substrate serotonin into *N*-acetylserotonin in the chloroplast and 5-methoxytryptamine in the cytoplasm. These two intermediates are then converted to melatonin by ASMT in the cytoplasm or SNAT in the chloroplast [72,73]. Interestingly, COMT, an enzyme that is similar to ASMT, was reported to play a pivotal role in the synthesis of phyto-melatonin, specifically existing in the cytoplasm of plants [65,74].

Compared to biosynthesis, limited information is available about phyto-melatonin catabolism. Several metabolites of melatonin have been detected in plants, including 2-hydroxymelatonin, cyclic-3-hydroxymelatonin, and AFMK [13]. These metabolites are formed through either enzymatic process or interactions between melatonin and ROS (Figure 1). AFMK, the first detected phyto-melatonin metabolite in water hyacinth (*Eichhornia crassipes*), is thought to produce via the catalysis of indoleamine 2,3-dioxygenase (IDO) [75,76,77]. AFMK can be further converted into AMK in animals and it is considered to exist in plants as well [13]. Furthermore, melatonin can be hydroxylated by members of 2-oxoglutarate-dependent dioxygenase (2-ODD) superfamily, among which 2-ODD 11, 19, 21, and 33 catalyzed the formation of 2-hydroxymelatonin [76] and 2-ODD 11, 26, and 33 catalyzed the conversion to cyclic-3-hydroxymelatonin in *Arabidopsis* [77]. Melatonin also can directly interact with ROS, further generating 2-hydroxymelatonin, cyclic-3-hydroxymelatonin, or AFMK [11]. The conversion of 2-hydroxymelatonin and cyclic-3-hydroxymelatonin to other unidentified products has not been reported in plants, however, the possibility cannot be excluded. More studies are needed to gain a better understanding of the mechanism of melatonin catabolism in plants.

## 3. Stress-Induced Melatonin Accumulation

It is widely reported that the production of phyto-melatonin is enhanced under different stresses, including heat, cold, drought, salinity, oxidation, heavy metal, or pathogen invasion [1,78], demonstrating that the molecule functions in the stress response. Phyto-melatonin accumulation is relatively closely associated with the gene expression and enzymic activity of the candidates lying on the biosynthesis and catabolism pathway of the melatonin under stress. For instance, the expression of melatonin synthetases (*TDC*, *T5H*, and *ASMT*) closely related to melatonin production in rice under excess Cd [44]. Besides, high temperature modulated the enzymic activities of SNAT and ASMT and further increased melatonin production in rice [79]. However, little is known about the pathway regulation mechanism of the production of melatonin in the response to stress. Recently, a transcription factor (*HsfA1a*) was found to directly bind to the COMT1 gene promoter under Cd stress, and then increase the concentration of melatonin in tomatoes [47].

Generally, the divergence of molecules concentrations closely connects with precursor availability [1]. In contrast to expectation, a serotonin boost in the biosynthesis pathway of melatonin is not linked with a significant rise in melatonin due to the lower catalytic efficiency of ASMT/COMT and SNAT when compared to that of TDC and T5H [80]. Given the low enzyme activity, previous studies mainly through modulating the expression of SNAT/ASMT from plants or HIOMT (the homologous gene of *ASMT* in animals) to analysis the role of endogenous phyto-melatonin exposed to stress, further confirming that melatonin confers plant stress tolerance (Table 2). In addition, serotonin seems to play the same role in defense against stress under cold condition in rice [81]. Similarly, the higher levels of 2-hydroxymelatonin suffered from a combination of cold and drought in rice suggested its potential functions in resisting stresses [76,77].

## 4. Melatonin, its Precursors and Metabolites Conferring Plant Abiotic Stress Resistance

**Figure 2:**
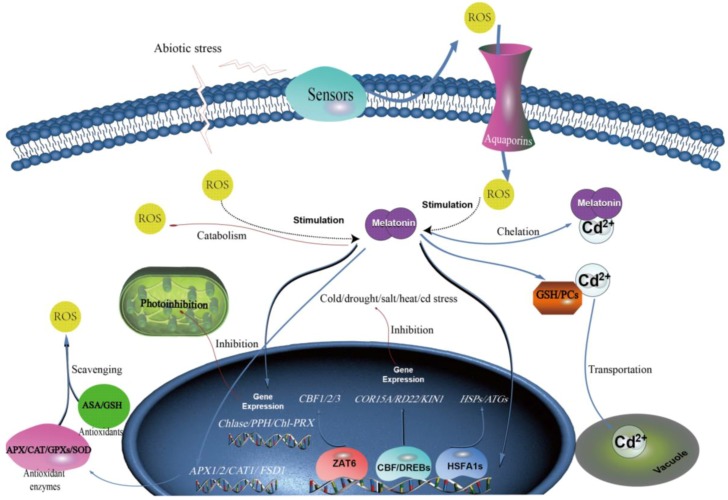
Melatonin-mediated abiotic stress response in plants. Abbreviation: ROS, reactive oxygen species; GSH, glutathione; PCs, phytochelatins; Cd, cadmium; SOD, superoxide dismutase; APX, ascorbate peroxidase; CAT, catalase; GPX, glutathione peroxidase; ASA, antioxidants ascorbic acid; GSH, glutathione. Dotted arrows represent the hypothetical pathway.

Under abiotic stress, there are two major sources of ROS generating at apoplast (signaling ROS) and cellular compartments, including chloroplast, peroxisome, and mitochondria (metabolic ROS) [87]. Metabolic ROS together with signaling ROS moving into the cytoplasm via aquaporins up-regulates melatonin production [88,89,90]. However, an excess of ROS leads to lipid peroxidation in cellular membranes, DNA damage, protein denaturation, carbohydrate oxidation, pigment breakdown, and impaired enzyme activity [91]. Therefore, plants have to maintain a balance between ROS generation and ROS scavenging under stress. Phyto-melatonin is one of the key ways to clear excessive ROS and cope with kinds of abiotic stress with other measures (Figure 2).

Melatonin can directly scavenge ROS and then produce at least three metabolites (2-hydroxymelatonin, cyclic-3-hydroxymelatonin, and AFMK). It has the higher capacity to scavenge ROS than the classic antioxidants, such as including vitamin C, vitamin E, glutathione, and NADH [11,92,93,94]. Expect clearing ROS, melatonin also can directly bind to several toxic metals to suppress damage formation [95]. Exogenous melatonin significantly decreased the accumulation of vanadium in *Citrullus lanatus*, and cadmium in rice and *Arabidopsis*, and further reducing the heavy metal stress [46,49,50].

In addition to directly interacting with ROS, melatonin can also activate the plant’s antioxidant system. ROS-scavenging enzyme systems, such as superoxide dismutase (SOD), ascorbate peroxidase (APX), catalase (CAT), glutathione peroxidase (GPX), the antioxidants ascorbic acid (ASA), glutathione (GSH), and tocopherol play an important role in plant stress response [96,97]. Exposed to various stresses, melatonin usually up-regulates the content of SOD, APX, CAT, and GPX in plants by regulating antioxidant-related genes expression. For instance, melatonin induced expression of *CAT1*, *APX1*, and *Fe-SOD* under high temperature in *Solanum lycopersicum* [28]. Melatonin was also reported to increase the activities of APX, CAT, and SOD by up-regulating *APX1/2*, *CAT1*, and *FSD1* transcripts in *Arabidopsis* in response to salt stress [31]. The relative expressions of several genes that are responsible for SOD, APX, and GPX were augmented in melatonin-treated seedlings exposed to vanadium stress in watermelon [50]. Furthermore, melatonin could activate the ASA-GSH cycle, an important antioxidant system in higher plants, to protect against abiotic stress. Under drought stress, the increased enzyme activity and expression of APX, dehydroascorbate reductase (DHAR), and monodehydroascorbate reductase (MDHAR) were responsible for melatonin-mediated increased GSH/(GSH + GSSG) and AsA/(ASA + DHA) in wheat seedlings [42]. GSH and ASA were substantially up-regulated as well in melatonin-treated tomato under cold stress [19]. Phyto-melatonin also enhanced contents of GSH and phytochelatins (PCs) in tomatoes under cadmium (Cd) stress, and then ATP-binding cassette transporters actively transported Cd-PCs and Cd-GSH complexes into the vacuole [47], contributing to heavy metal stress resistance by mediating sequestration or chelation [51].

Photosynthesis is highly sensitive to temperature, drought, salt, and heavy metal, and usually suppressed when exposed to these stresses [98]. Melatonin can enhance chlorophyll contention, electron transport, and stomatal conductance to alleviate photosynthetic inhibition that is caused by stress [9,10,11]. By down-regulating the chlorophyll degradation genes (chlorophyllase (*Chase*), pheophytinase (*PPH*) and Chl-degrading peroxidase (*Chl-PRX*)), melatonin protects chlorophyll of plants from various stresses [21,34,40,51,99]. Melatonin application also regulated electron transport system, such as improving nonphotochemical quenching (NPQ) or photochemical quenching (qP), and further increased the maximal quantum yield of PSII photochemistry (Fv/Fm) [34,41]. Furthermore, exogenous melatonin raised stomatal conductance to relieve the limitation of CO_2_ that is caused by drought [40].

Transcription factors regulation is one of the critical ways of the phyto-melatonin-mediated stress response. Three melatonin-mediated abiotic stress transcription factors (Zinc Finger protein 6 (*ZAT6*), Heat Shock Factors (*HSFA1s*), and C-Repeat-Binding Factor (*CBF*)/Drought Response Element Binding 1 Factors (*DREB1s*)) were detected in plants. Melatonin up-regulated *ZAT6* expression, which activated the *CBF* pathway and further mediated the freezing stress response [78]. *HSFA1s*, induced by melatonin, could up-regulate the transcription levels of the heat-response genes *HSFA2*, *HSA32*, *HSP90*, and *HSP101*, further conferring thermotolerance and Cd tolerance [26,47]. Meanwhile, the up-regulation of *CBF*/*DREB1s* closely associated with high level of melatonin led to an increase in the transcription levels of multiple stress-responsive genes (cold-related 15A (*COR15A*), responsive to dehydration 22 (*RD22*), and cold-inducible 1 (*KIN1*)), resulting in improved resistance to salt, drought, and freezing stresses [100]. Transcription factors activated by melatonin under abiotic stress play important roles in regulating the transcription of stress-responsive genes. Notably, 2-hydroxymelatonin, the metabolite of melatonin, also up-regulated the transcription factors *Myb4* and *AP37* to alleviate the effects of multiple abiotic stresses in rice [64].

Similar to melatonin, related intermediates and metabolites can also initiate plant stress response. Tryptophan, the primary precursor of phyto-melatonin, is also the substrate for auxin, glucosinolates, phytoalexins, alkaloids, and indoles, therefore it is an important molecule for the plant stress response [101,102]. For tryptamine, it is closely related with the light-enhanced resistance in rice [103]. Serotonin was reported to enhance a diverse range of abiotic stress responses, including improving survival under salinity by mediating the influx of ions into chloroplasts [104], enhancing the heavy metal tolerance through the high capacity for binding cadmium to form stable complexes [8], and relieving X-ray radiation stress [62]. It is worth noting that 2-hydroxymelatonin was amplified in rice in response to cold and drought stress [64]. The role of *N*-acetylserotonin in plants abiotic stress has not been identified, but *N*-acetylserotonin exhibited antioxidant activity in animals [105]. The involvement of 5-methoxytryptamine, cyclic-3-hydroxymelatonin, and AFMK in the enhancement of plant stress resistance remains un-explored until now, but may potentially contribute to stress response based upon their molecular similarity with melatonin.

## 5. Melatonin and Its Precursors and Metabolites Play Key Roles in Plant Biotic Stress

**Figure 3:**
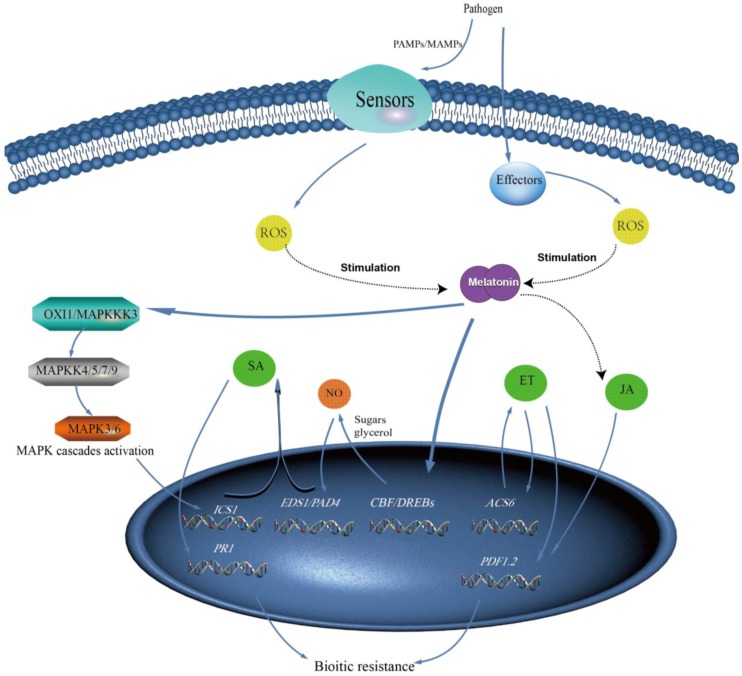
Melatonin-mediated biotic stress response in plants. Abbreviation: ROS, reactive oxygen species; PAMPs, pathogen-associated molecular patterns; MAMPs, microbe-associated molecular patterns; MAPK, Mitogen-activated protein kinase; NO, nitric oxide; SA, salicylic acid; JA, jasmonic acid; ET, ethylene. Dotted arrows represent the hypothetical pathway.

Plants have evolved a melatonin immune system to protect individual cells against pathogen infection (Figure 3). Pathogen invasion induces plants to produce ROS by effectors or pathogen-microbe-associated molecular patterns (PAMPs or MAMPs) [106,107]. Similar to abiotic stress, ROS leading to the up-regulation of melatonin is observed upon infection by pathogens, however, the mechanism is not yet clear [54]. The role of melatonin in defense against pathogen attacks has been investigated in terms of the signaling pathway and mechanisms that are involved, among which the interaction of melatonin with salicylic acid (SA) is particularly important [54]. SA is an important defense hormone that is involved in the innate plant immunity [10], and it increases the transcript levels of a defense-gene (pathogenesis-related 1 (*PR1*)) by a receptor nonexpressor of PR1 (*NPR1*). High level of melatonin could indirectly induce transcription of isochorismate synthase 1 (*ICS1*), which is responsible for the biosynthesis of SA, by stimulating the mitogen-activated protein kinase (MAPK) cascade (MAPKKK3/OXI1-MAPKK4/5/7/9-MAPK3/6) in *Arabidopsis thaliana* infected with *Pseudomonas syringe* pv. Tomato (Pst) DC3000 [52,54]. Besides MAPKs, nitric oxide (NO) also induces the innate plant immunity via positively modulating the expression levels of both SA synthesis genes (*AtEDS1* and *AtPAD4*) and downstream SA resistant genes (*AtPR1*, *AtPR2*, and *AtPR5*) [53,108]. Intriguingly, melatonin induced augmented the transcription of *CBF*/*DREB1s*, leading to an increase in NO by enhancing the accumulation of soluble sugars in *Arabidopsis thaliana* infected with *Pst* DC3000 [100]. Ethylene (ET) and jasmonic acid (JA) are involved in melatonin-mediated disease resistance as well [109]. For example, melatonin up-regulated 1-aminocyclopropane-1-carboxylate synthase 6 (*ACS6*), which is a key enzyme in the biosynthesis of ET, and then induced expression of an antimicrobial peptide (plant defensin 1.2 (*PDF1.2*)) via ethylene insensitive 2 (*EIN2*) [52]. As JA could induce the expression of *PDF1.2* as well as melatonin, we cannot rule out the possible involvement of JA with melatonin in the pathogen resistance pathway [52]. Additionally, *MaHSP90s* was reported to be up-regulated by melatonin triggered the effects of defense-related plant hormones (IAA, SA, JA, and ET) [56]. It is notable that transcriptome data analysis of melatonin-treated watermelon and *Arabidopsis* showed that various defense-related genes that were involved in plant hormone signaling or innate plant immunity, and the further analysis could lead to deep insight in molecular mechanisms of pathogen resistance for plants treated with melatonin [110,111].

Similar to melatonin, serotonin may also be essential in long distance and rapid signaling in response to pathogen attacks by mediating ROS and interacting with hormone signaling networks [16]. The indole alkaloid tryptamine, a key factor in light-enhanced resistance, inhibited infection by *Magnaporthe oryzae* in rice [60]. 2-hydroxymelatonin and *N*-acetylserotonin could also activate MAPKs to confer the biotic stress, but to a lesser degree than melatonin in *Arabidopsis thaliana* [63].

## 6. Conclusions

Faced with environmental changes, melatonin biosynthesis, and catabolism pathway would take essential functions in plants for coping with various stresses. Phyto-melatonin with its precursors and metabolites were adjusted to mitigate abiotic stress through both direct (scavenging ROS and chelating heavy metal) and indirect (activating the plant’s antioxidant system, transporting heavy metal, alleviating photosynthesis inhibition, and regulating transcription factor) mechanisms. Moreover, melatonin imposes plant anti-pathogenic functions by activation of plant stress-relevant hormones, such as SA, ET, or JA. For a long time, previous studies have focused on how melatonin increases the abiotic and biotic stress resistance of plants. However, little attention has been given to the compounds located in the synthesis and catabolism pathway of melatonin. Further investigations on the role of precursors and metabolites of melatonin will shed more light on the underlying plant stress resistance.

## Figures and Tables

**Table 1:** Melatonin and its precursors and metabolites mediating plant stress resistance.

Compounds	Stresses	Plant Species
melatonin	cold	*Arabidopsis thaliana* [17,18], *Solanum lycopersicum* [19,20], rice [21], *Prunus persica* [22], *Citrullus lanatus* [23], *Triticum aestivum* [24], and *Cucumis sativus* [25]
melatonin	heat	*Arabidopsis thaliana* [26], *Solanum lycopersicum* [27,28], *Lolium perenne* [29], and *Festuca arundinacea* [30]
melatonin	salt	*Arabidopsis thaliana* [31,32], *Cucumis sativus* [33], *Citrullus lanatus* [34], *Helianthus annuus* [35], and *Zea mays* [36]
melatonin	drought	*Arabidopsis thaliana* [37], *Malus zumi* [38], *Solanum lycopersicum* [39], *Zea mays* [40,41], *Triticum aestivum* [42], and *Medicago sativa* [43]
melatonin	heavy metal	rice [44,45,46], *Solanum lycopersicum* [47,48], *Medicago sativa* [49], *Citrullus lanatus* [50], and wheat [51]
melatonin	pathogen	*Arabidopsis thaliana* [52,53,54], rice [55], *Musa acuminate* [56], potato [57], cassava [58], and *Malus pumila* [59]
tryptamine	pathogen	rice [60]
serotonin	salt	*Helianthus annuus* [61]
Serotonin	radiation	*Vicia faba* [62]
Serotonin	heavy metal	rice [44]
*N*-acetylserotonin	pathogen	*Arabidopsis thaliana* [63]
2-hydroxymelatonin	combination of cold and drought	rice [64]
2-hydroxymelatonin	pathogen	*Arabidopsis thaliana* [63]

**Table 2:** Melatonin-related transgenic plants under stress.

Genetically Modified Plants	Melatonin Level (↑up↓down)	Stress Resistance
human *SNAT*/*HIOMT* overexpressed in *Nicotiana sylvestris* [82]	↑	increased resistance to UV-B radiation
human *SNAT* overexpressed in transgenic rice [81]	↑	increased cold resistance
Sheep *HIOMT* overexpressed in micro-tom tomato [83]	↑	increased resistance to drought
*SNAT* knockout mutant *Arabidopsis* [84]	↓	increased the susceptibility to avirulent pathogen
suppression of *SNAT*/*ASMT* in rice [85]	↓	increased the abiotic stress susceptibility
maize *ASMT* overexpressed in *Arabidopsis* [37]	↑	enhanced drought tolerance
tomato *ASMT* overexpressed in tomato [27]	↑	enhanced thermotolerance
*SNAT* knockout mutant *Arabidopsis* [31]	↓	decreased salinity tolerance
ovine *AANAT/HIOMT* overexpressed in switchgrass [86]	↑	improved salt-tolerance
rice *SNAT* overexpressed in rice [46]	↑	conferred resistance to cadmium
alfalfa *SNAT* overexpressed in *Arabidopsis* [49]	↑	conferred plant tolerance against cadmium

## Abbreviations

AFMK	*N*^1^-acetyl-*N*^2^-formyl-5-methoxyknuramine
AMK	*N*-acetyl-5-methoxyknuramine
2-ODD	2-oxoglutarate-dependent dioxygenase
ROS	reactive oxygen species
SA	salicylic acid
ET	ethylene
JA	jasmonic acid
TPH	tryptophan hydroxylase
TDC	tryptophan decarboxylase
T5H	tryptamine 5-hydroxylase
SNAT	serotonin *N*-acetyltransferase
ASMT	*N*-acetylserotonin methyltransferase
COMT	caffeic acid *O*-methyltransferase
AMK	*N*-acetyl-5-methoxyknuramine
HIMOT	hydroxyindole-*O*-methyltransferase
Cd	cadmium
IDO	indoleamine 2,3-dioxygenase
SOD	superoxide dismutase
APX	ascorbate peroxidase
CAT	catalase
GPX	glutathione peroxidase
ASA	antioxidants ascorbic acid
GSH	glutathione
DHAR	dehydroascorbate reductase
MDHAR	monodehydroascorbate reductase
Cd	cadmium
Fv/Fm	maximal quantum yield of PSII photochemistry
PCs	phytochelatins
qP	photochemical quenching
NPQ	nonphotochemical quenching
PAMPs	pathogen-associated molecular patterns
MAMPs	microbe-associated molecular patterns
